# Opto-Current-Clamp Actuation of Cortical Neurons Using a Strategically Designed Channelrhodopsin

**DOI:** 10.1371/journal.pone.0012893

**Published:** 2010-09-23

**Authors:** Lei Wen, Hongxia Wang, Saki Tanimoto, Ryo Egawa, Yoshiya Matsuzaka, Hajime Mushiake, Toru Ishizuka, Hiromu Yawo

**Affiliations:** 1 Department of Developmental Biology and Neuroscience, Tohoku University Graduate School of Life Sciences, Sendai, Japan; 2 Japan Science and Technology Agency (JST), Core Research of Evolutional Science & Technology (CREST), Tokyo, Japan; 3 Tohoku University Basic and Translational Research Center for Global Brain Science, Sendai, Japan; 4 Department of Physiology, Tohoku University Graduate School of Medicine, Sendai, Japan; 5 Center for Neuroscience, Tohoku University Graduate School of Medicine, Sendai, Japan; Mount Sinai School of Medicine, United States of America

## Abstract

**Background:**

Optogenetic manipulation of a neuronal network enables one to reveal how high-order functions emerge in the central nervous system. One of the *Chlamydomonas* rhodopsins, channelrhodopsin-1 (ChR1), has several advantages over channelrhodopsin-2 (ChR2) in terms of the photocurrent kinetics. Improved temporal resolution would be expected by the optogenetics using the ChR1 variants with enhanced photocurrents.

**Methodology/Principal Findings:**

The photocurrent retardation of ChR1 was overcome by exchanging the sixth helix domain with its counterpart in ChR2 producing Channelrhodopsin-green receiver (ChRGR) with further reform of the molecule. When the ChRGR photocurrent was measured from the expressing HEK293 cells under whole-cell patch clamp, it was preferentially activated by green light and has fast kinetics with minimal desensitization. With its kinetic advantages the use of ChRGR would enable one to inject a current into a neuron by the time course as predicted by the intensity of the shedding light (opto-current clamp). The ChRGR was also expressed in the motor cortical neurons of a mouse using Sindbis pseudovirion vectors. When an oscillatory LED light signal was applied sweeping through frequencies, it robustly evoked action potentials synchronized to the oscillatory light at 5–10 Hz in layer 5 pyramidal cells in the cortical slice. The ChRGR-expressing neurons were also driven *in vivo* with monitoring local field potentials (LFPs) and the time-frequency energy distribution of the light-evoked response was investigated using wavelet analysis. The oscillatory light enhanced both the in-phase and out-phase responses of LFP at the preferential frequencies of 5–10 Hz. The spread of activity was evidenced by the fact that there were many c-Fos-immunoreactive neurons that were negative for ChRGR in a region of the motor cortex.

**Conclusions/Significance:**

The opto-current-clamp study suggests that the depolarization of a small number of neurons wakes up the motor cortical network over some critical point to the activated state.

## Introduction

One of the enigmas in neuroscience is how high-order neural functions emerge from network communications among many neurons of various traits. To reveal this optical stimulating and recording methods are advantageous because of their high spatial and temporal resolutions [1], [2]. Recently the optogenetics, which endows neurons with photosensitivity by genetic engineering methods, has opened new horizon in neural sciences [3]–[5].

Channelrhodopsin-1 (ChR1) and -2 (ChR2), which are involved in the light-dependent behavior of a unicellular green alga, *Chlamydomonas reinhardtii*, are unique in the family of archaeal-type rhodopsins [6]–[9], since each works as both photoreceptor and ion channel. Recently ChR2-mediated photostimulation of neurons has been applied to investigate the function of neural networks *in vivo* [10]–[13]. ChR2 can selectively activate neurons and is able to modify neuronal circuits by inducing synaptic plasticity. Moreover, ChR2-expressing transgenic animals have been generated and successfully used to study the neural basis of behavioral responses in *Caenorhabditis elegans* [14], zebrafish [15] and mammals [16], [17]. Given its superiority in spatio-temporal resolution, ChR2 has become a powerful tool for the investigation of neural networks of various animals. ChR2 may also have potential as a visual prosthesis for photoreceptor degeneration [17]. In retinal degenerative diseases such as retinitis pigmentosa, photoreceptor cells are lost while the inner retinal neurons such as retinal ganglion cells and bipolar cells are preserved. Exogenous expression of ChR2 in these neurons using viral vectors or *in vivo* electroporation methods restores visually-evoked responses in the visual cortex of rodents [18]–[20]. However, there are still some technological issues that need to be addressed to achieve the maximum potential of channelrhodopsins. First, photosensitive channels with various wavelength sensitivities would be desirable. Second, the prominent desensitization of ChR2 photocurrent limits its application for repetitive stimulation at high frequency. Third, the fast turning-on (ON) and -off (OFF) kinetics are ideal for manipulating membrane potential by light.

To manipulate optogenetically the neuronal activity, the ChR1 photocurrent has several advantages over the ChR2, such as the small desensitization and the rapid ON and OFF kinetics [8], [9], [21]–[23]. Unfortunately, it is small in amplitude because of the retarded membrane expression and other unresolved reasons [23], [24]. Here we found that this retardation could be overcome by exchanging the sixth helix domain with its counterpart in ChR2 producing channelrhodopsin-green receiver (ChRGR) with further reform of the molecule. Since ChRGR showed smaller desensitization than ChR1 and the fast ON and OFF kinetics, it can be optimized for optogenetic injection of the patterned current into a neuron (opto-current clamp).

## Results

**Figure 1:**
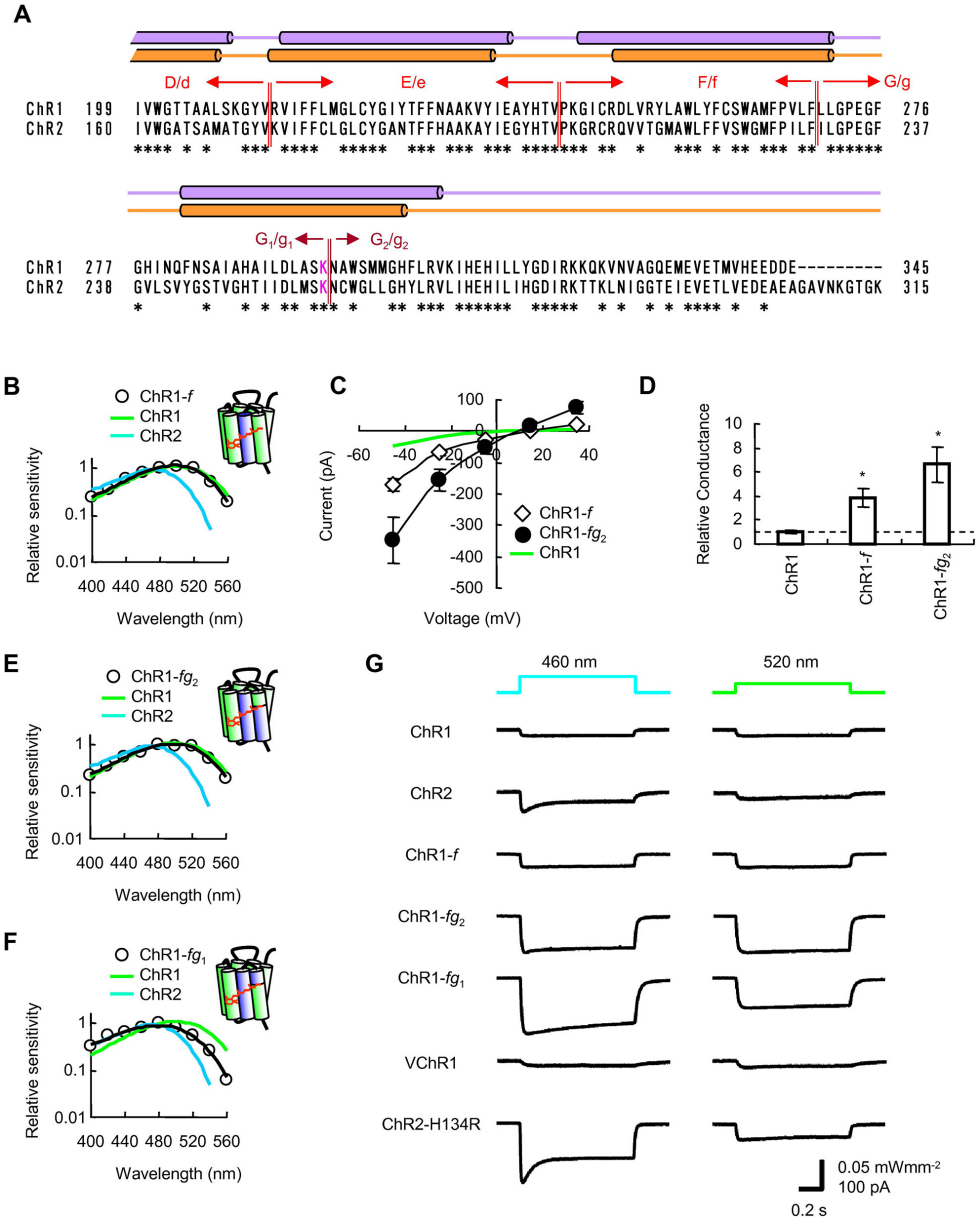
Strategic design of channelrhodopsin-green receiver. **A**. Sequence alignment of ChR1(199–345) and ChR2 (160–315). The identical amino acids are indicated with an asterisk (*). The putative fifth to seventh transmembrane domains, E/e to G/g, and subdomains are indicated. The putative transmembrane helixes are indicated by purple cylinders [24], [25] or orange cylinders [7]. **B**. Action spectrum of ChR1-*f* photocurrent in comparison with ChR1 and 2. **C**. Photocurrent-voltage relationship. **D**. Summary of conductance relative to ChR1. Asterisks indicate statistical significance (*P*<0.005, Mann-Whitney *U*-test). **E** and **F**. Action spectrum of ChR1-*fg*_2_ (E) and ChR1-*fg*_1_ (F) photocurrents. **G**. Each trace is an average photocurrent evoked at either 460 or 520 nm. The number of samples are, 8 (ChR1), 8 (ChR2), 8 (ChR1-*f*), 8 (ChR1-*fg*_2_), 8 (ChR1-*fg*_1_), 6 (VChR1) or 9 (ChR2-H134R).

It has been suggested that the channel conductance or the probability of a channel being open is likely to be dependent on the sixth transmembrane helix of ChRs [23]. To test this, the sixth transmembrane domain (“*F*”, Figure 1A) of ChR1 was replaced by its counterpart (“*f*”) from ChR2 producing a chimera, ChR1-*f*. When expressed in HEK293 cells, this replacement enhanced the whole-cell conductance without changing the reversal potential and the action spectrum (Figure 1B–1C). In the middle of the seventh transmembrane helix of ChR1, Lys^296^ is where the retinal is covalently binding. We subdivided the seventh transmembrane domain (“*G*”) into two subdomains; one from Leu^270^ to Lys^296^ (“*G*_1_”) and the other from Asn^297^ to Glu^345^ ( “*G*_2_”), then replaced each subdomain with its counterpart from ChR2 (“*g*”), *g*_1_ (Ile^231^-Lys^257^) or *g*_2_ (Asn^258^-Lys^315^). Each chimera channelrhodopsin, ChR1-*fg*_1_ or ChR1-*fg*_2_, was expressed in HEK293 cells and the action spectrum was again investigated. As shown in Figure 1E, the action spectrum of ChR1-*fg*_2_ was almost identical to that of ChR1, whereas that of ChR1-*fg*_1_ was blue-shifted (Figure 1F). The effective conductance of ChR1-*fg*_2_ was 0.29±0.06 µS/pF (n = 10), as about 7-fold that of ChR1 (Figure 1C, 1D). Since the photocurrent size was variable from cell to cell, the averaged photocurrent was compared at 460 (0.027 mWmm^−2^) and 520 nm (0.015 mWmm^−2^). The photocurrent of ChR1-*fg*_2_ was large at both wavelengths as expected from its effective conductance and the action spectrum (Figure 1G). In response to green light it generated relatively large photocurrents if compared to ChR2 or its gain-of-function variant ChR2-H134R [14], whereas in response to blue light their peak amplitudes were almost similar (Figure S1). Recently, one of channelrhodopsins derived from *Volvox* (VChR1) has been shown to have a red-shifted action spectrum [25]. Although its photocurrent is near maximal at 520 nm, the average photocurrent (−29±6 pA, n = 6) was smaller than that of ChR1-*fg*_2_ (−146±38 pA, n = 8) with a significant difference (Figure S1).

**Figure 2:**
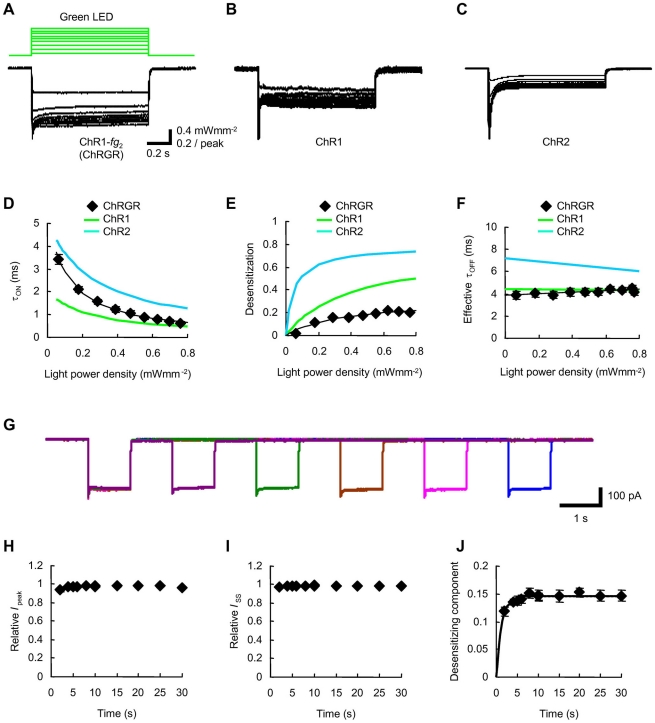
Kinetic profiles of photocurrents. **A–C**. Photocurrent traces of ChR1-*fg*_2_ (ChRGR, A), ChR1 (B) and ChR2 (C) evoked by green LED with various strengths (505±15 nm, 1 s pulse, 0.064–0.77 mWmm^−2^). All experiments were done at 34°C. Each photocurrent was normalized to the peak value evoked by the maximal level. **D–F**. Parameters of photocurrent kinetics. All experiments were done at 34°C. (D) The apparent ON time constant (τ_ON_)-light power density (*L*) relationships. Each relationship was fitted to a function, τ_ON_ = (*a*+*bL*)^−1^ [28]. (E) The desensitization-light power density relationships. Each relationship was fitted to a Michaelis-Menten-type kinetics [22]. (F) The effective OFF time constant (τ_OFF_)-light power density relationships. Each relationship was fitted to a first order function. **G**. Typical traces of ChRGR photocurrents in response to double light pulse stimulation by green LED (505±15 nm, 1 s pulse, 0.77 mWmm^−2^). **H**. The recovery time course of the peak current (*I*_peak_). **I**. The recovery time course of the steady-state current (*I*_ss_). **J**. The recovery time course of the desensitizing component. The process was fitted to a single exponential relationship (time constant, 1.3 s).

Since the ChR1-*fg*_2_, which we named channelrhodopsin-green receiver (ChRGR), generated photocurrents of relatively large amplitudes, it is a potential candidate of the optogenetic actuator by green light. To test this possibility, its photocurrent kinetics was investigated. Because the photocurrent kinetics are dependent on the light power density, the holding potential as well as the temperature, each photocurrent was measured at the holding potential of −40 mV and at 34°C, with various levels of light power density of green LED (505±15 nm, 1 s pulse, 0.064–0.77 mWmm^−2^) (Figure 2A). The kinetic profile of ChRGR photocurrent was characterized by the ON kinetics, the desensitization and the OFF kinetics under comparison to those of ChR1 or 2 (Figure 2B, 2C). Quantitatively, the photocurrent ON kinetics, which followed a single exponential function, was evaluated by its apparent time constant (τ_ON_) as a function of the light power density (Figure 2D). The difference between the peak photocurrent and the steady-state photocurrent at the end of 1s pulse was divided by the peak photocurrent amplitude, and the desensitization was evaluated as a function of the light power density (Figure 2E). Since the photocurrent OFF kinetics was best fitted by two exponential functions [23], it was evaluated by the effective OFF time constant (τ_OFF_), which is the time to reach *e* ^−1^ (37%) of the steady-state amplitude (Figure 2F). The photocurrents of ChRGR were rather similar to those of ChR1 than to those of ChR2 in their kinetic profiles. The recovery from the desensitization was examined by applying two 1 s light pulse (0.77 mWmm^−2^) with variable intervals (Figure 2G). Both the peak and steady-state current was only little influenced by the preceding light exposure (Figure 2H and 2I) and the relatively rapid recovery of desensitized component with a time constant of 1.3 s (Figure 2J).

**Figure 3:**
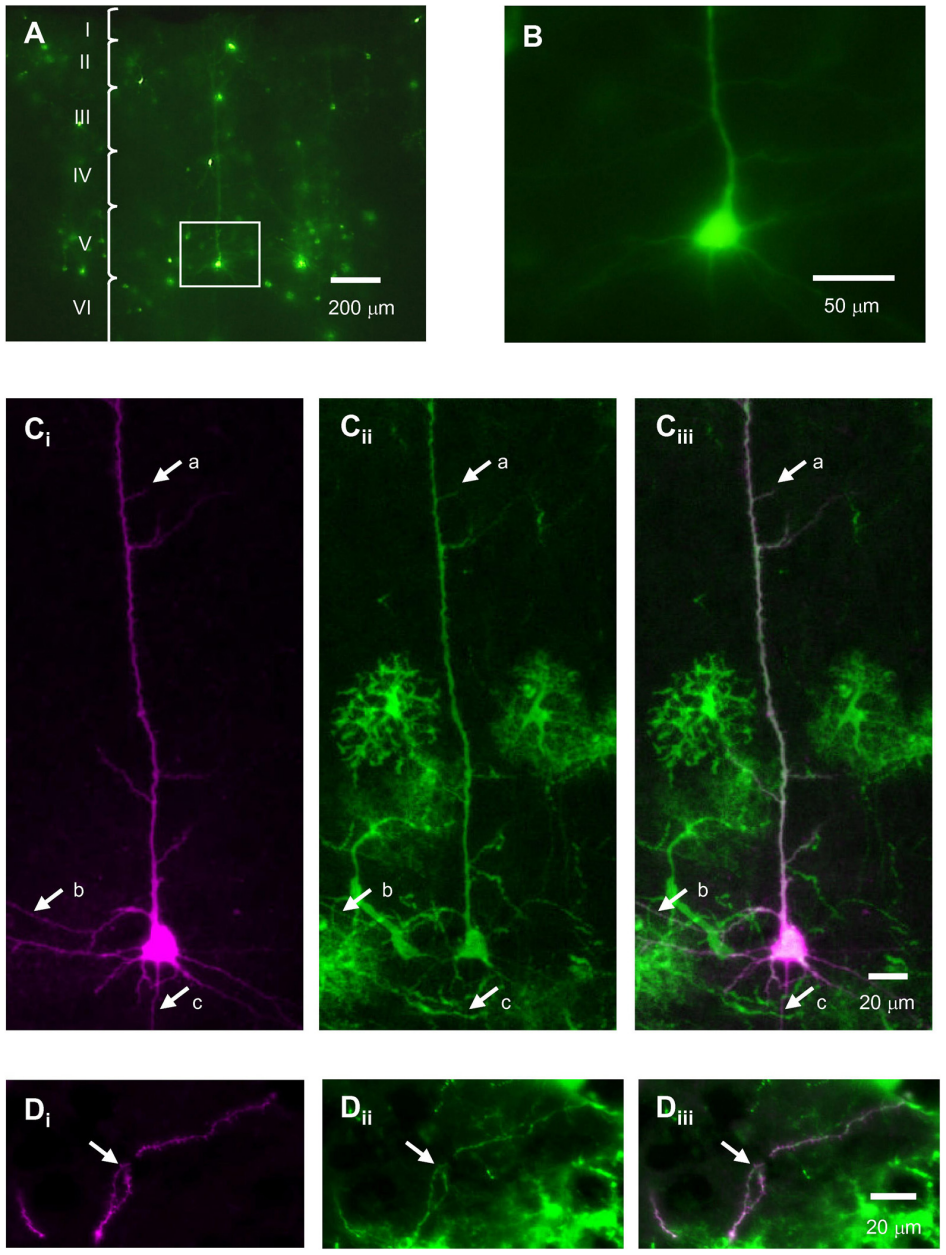
Neuronal expression. **A**. Neurons expressing ChRGR-Venus in a cortical slice. **B**. A typical L5 pyramidal neuron shown in A. **C**. Morphology of biocytin-filled neuron (**C_i_**), its expression of ChRGR-Venus (**C_ii_**) and the merged image of C_i_ and C_ii_ (**C_iii_**). The arrows indicate the apical dendrite and its branches (a), the basal dendrites (b) and the axon (c). **D**. Morphology of tufted dendrites (indicated by an arrow) of a L5 pyramidal neuron in cortical layer I (**D_i_**), its expression of ChRGR-Venus (**D_ii_**) and the merged image of D_i_ and D_ii_ (**D_iii_**).

To manipulate optogenetically the neuronal activity the ChRGR has several advantages such as; (i) the small desensitization, (ii) the rapid recovery from desensitization, (iii) the rapid ON and OFF kinetics and (iv) the relatively large photocurrent evoked by light of long wavelength. These properties enable one to inject a current into a neuron by the time course as predicted by the intensity of the shedding light. That is, when the light is given in a square-pulse, a square-pulse current can be expected to be induced. When the light is given in a sinusoidal wave, a sinusoidal current can be expected to be induced. These optically induced currents would generate the membrane potential responses that are dependent on the neuron's membrane properties such as the membrane resistance, the membrane capacitance and the types of ion channels (opto-current-clamp). The membrane potential response is also dependent on the neuron's morphology, the distribution of intrinsic ion channels and the effective localization of ChRGR. We tested the opto-current-clamp using a ChRGR-expressing layer 5 (L5) pyramidal neuron of the mouse motor cortex. Neurons expressing ChRGR-Venus were found in layer II–VI of the cortex in 12 hours after injecting Sindbis pseudovirion vectors, were normal in appearance and their soma, dendrites and axons were fluorescently labeled (Figure 3A). One of the ChRGR-Venus-expressing L5 pyramidal neurons was identified in an acute slice of the cerebral cortex by its location, shape and size (Figure 3B). The fine processes such as axon, apical and basal dendrites, which were identified by the intracellular administration of biocytin (Figure 3C_i_), co-expressed ChRGR-Venus (Figures 3C_ii_ and 3C_iii_). Fine tufted dendrites with spines were also identified in layer I (Figure 3D_i_). These dendrites were also distinctly co-expressing ChRGR-Venus (Figure 3D_ii_ and 3D_iii_). However, its expression was indistinct in the spine. The membrane properties of the ChRGR-expressing neurons were the same as the non-expressing ones (Table S1) and direct current injection through the patch electrode evoked repetitive neuronal firings with almost the same frequency-current relationship (Figure S2A and S2B).

**Figure 4:**
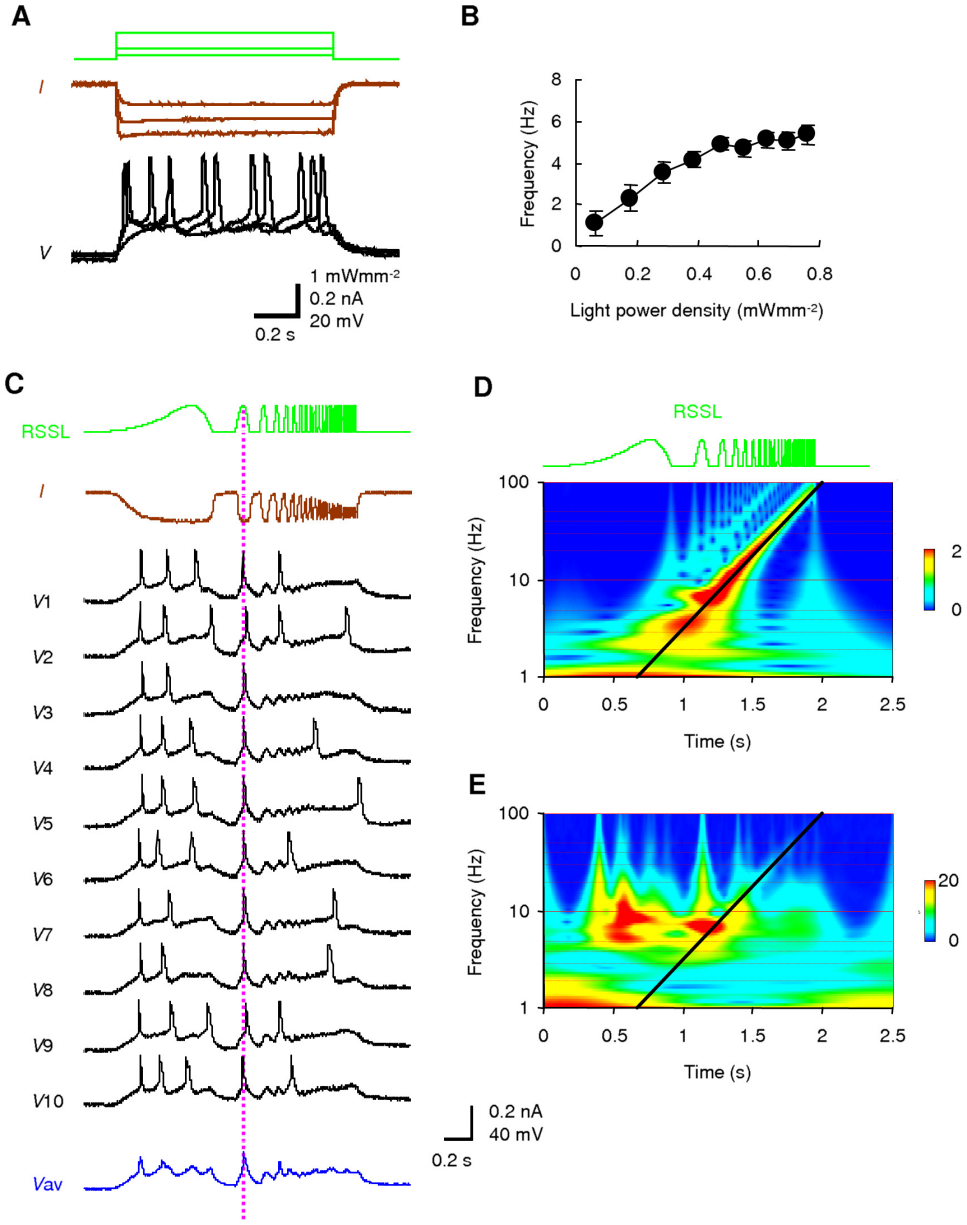
Opto-current clamp of a cortical neuron. **A**. Membrane currents under voltage clamp (brown traces) and potentials under current clamp (black traces) in response to the green LED pulses (green traces) applied on the whole visual field. **B**. Frequency of action potentials as a function of light power density. **C**. The rectified sinusoidal sweep of light (RSSL) from 0.1 to 100 Hz (top green), the photocurrent response of an L5 pyramidal neuron under voltage clamp (*I*, brown), the trial-to-trial responses of the membrane potential under current clamp (*V*1–*V*10, black) and the averaged membrane potential (*V*av, blue). **D**. Wavelet analysis of voltage-clamp response. The relative level of energy coefficient is indicated by the pseudocolor scaling. The line indicates the time-frequency relationship of the RSSL. **E**. Wavelet analysis of current-clamp response.

When a green LED pulse of 1-s duration was applied on the whole visual field, it evoked an inward current with minimal desensitization under voltage-clamp of an L5 pyramidal neuron (Figure 4A). Under current-clamp, it depolarized the neuron in a light power density-dependent manner (see also Figure S2C). Just above the threshold the neuron fired at 3–10 Hz with a regular interval (Figure 4A). Almost the same frequency-light power density relationship was observed in other L5 pyramidal neurons (n = 11) (Figure 4B). We next generated an oscillatory LED light signal that sweeps through frequencies between 0.1 and 100 Hz over 2s (Figure 4C, the top trace, the rectified sinusoidal sweep of light, RSSL) [26], [27]. Since both the ON and the OFF kinetics of the ChRGR photocurrent are fast, the RSSL was expected to evoke an oscillatory photocurrent. As shown in Figure 4C, trace *I* (see also Figure S3), an oscillatory photocurrent was actually evoked between 0.1 and 100 Hz, although the amplitude was reduced at high frequency. The waveform was also distorted as the photocurrent amplitude follows a non-linear relationship of the light power density [22], [28] (Figure S4). The RSSL is useful to investigate the input responsiveness of a neuron or a neuronal network. In the presence of 1 µM TTX, it oscillated the membrane potential between the resting potential and the depolarized potential at low frequency region (Figure S3, trace *V*) under current clamp. However, at high frequency region, the relative change of membrane potential was smaller than the relative change of photocurrent under voltage clamp (Figure S3) because of the large membrane time constant of L5 pyramidal neurons (50±11 ms, n = 6). In the absence of TTX, it robustly evoked action potentials within a narrow time window (Figure 4C, *V*1–*V*10). The robustness of a response to the oscillatory change of light power density was clearly demonstrated by applying wavelet analysis. If the response of an issue changes its amplitude in synchronous with the RSSL, the high energy coefficient values of wavelet analysis would be expected to distribute along the time-frequency relationship of the RSSL. Actually, the photocurrent response under voltage clamp followed the oscillatory change of light power density in a wide frequency range during RSSL (Figure 4D). On the other hand, the membrane potential response under current clamp response did not well follow the oscillatory change of light power density at a high-frequency region (Figure 4E). Rather, the energy coefficient value was relatively high at two time windows, one at the onset of slow depolarization and the other synchronized to the RSSL at 5–10 Hz. The robustness was also evaluated by the averaged current-clamp response (Figure 4C, *V*av). It peaked at 1.2 s from the onset, which corresponded to 6.3 Hz. The frequency evoking robust firing of L5 pyramidal neurons was on average 6.1±1.0 Hz (n = 11 neurons).

**Figure 5:**
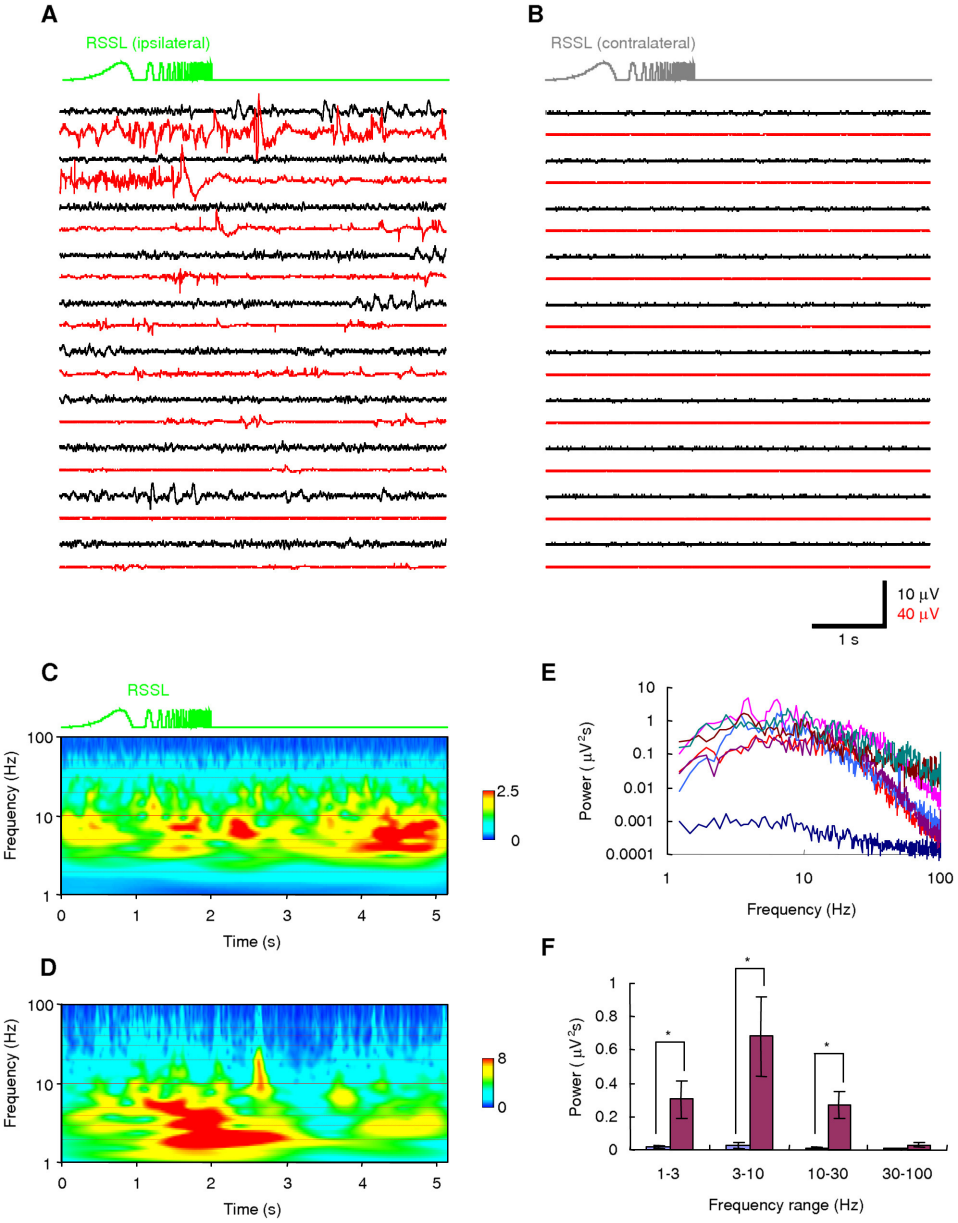
Opto-current clamp of the cortical network in living mice. **A**. The trial-to-trial responses of LFP (black lines) and EMG (red lines) under opto-current clamp by the RSSL. **B**. Control LFP and EMG traces by the RSSL on the contralateral cortex. **C**. Wavelet analysis of the LFP. **D**. Wavelet analysis of the EMG. **E**. Power spectra of opto-current-clamp responses of LFP from each animal. **F**. Summary of the LFP power spectrum analysis: control (contralateral RSSL, blue columns, left) and test (ipsilateral RSSL, crimson column, right). Asterisks indicate statistical significances (*P*<0.05, Wilcoxon signed-ranks test).

The ChR2-expressing neurons were shown previously to be activated by blue light even *in vivo* [10]–[20]. To examine whether ChRGR-expressing neurons are able to be activated *in vivo*, the local field potential (LFP) was recorded from the motor-cortical region close to the site where the Sindbis pseudovirion vectors were injected. The green LED light was focused on this site and reached the injected site through the dura mater (Figure 5A). The illumination to the contralateral non-injected site was used as a control (Figure 5B). The opto-current-clamp by RSSL evoked some LFP responses in the cortex. It also evoked electromyogram (EMG) responses in the contralateral lower limb muscles where the topographic projections of activated cortical neurons were expected. Each LFP response consisted of either positive-to-negative or negative-to-positive biphasic waves reminiscent of the population spikes of neurons. Sometimes, these LFP waves were accompanied by EMG waves. To find the frequency preferences, the wavelet analysis was applied to the LFP recordings. The frequency preference of the light-evoked responses was shown to be at 3–10 Hz (Figure 5C). Some LFP responses were evoked in-phase during the RSSL, whereas they were frequently evoked out-phase during the dark period. The RSSL also enhanced both the in-phase and out-phase responses of EMG (Figure 5D). In each animal the power spectrum was analyzed for the LFP responses (Figure 5E). In summary, the RSSL to the cortex enhanced the LFP activity preferentially at 3–10 Hz (Figure 5F).

**Figure 6:**
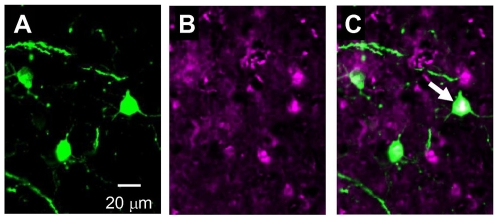
Spreading activity. **A–C**. The expression of c-Fos in the cortical neurons after a series of experiments; ChRGR-Venus (A), anti-c-Fos immunoreactivity (B) and merge (C). The white arrow indicates the ChRGR-expressing L5 pyramidal neuron which also expressed c-Fos at a relatively high level.

When a neuron is repetitively fired, the expression of some immediate early gene products such as Arc, c-Fos and Zif268 are enhanced through Ca^2+^-dependent mechanisms [29], [30], [31]. The expression of c-Fos was immunohistochemically identified after repetitive RSSL tests. Some of the ChRGR-expressing neurons also expressed c-Fos at a relatively high level but others did not (Figure 6A–6C and Figure S5). The expression of c-Fos was also enhanced distinctively in the cells that did not express ChRGR.

## Discussion

When some of the transmembrane helixes of ChR2 was replaced by the counterparts of ChR1 and produced chimera channelrhodopsins, the exchange distinctively affected photocurrent properties such as the wavelength sensitivity, conductance, desensitization and ON-OFF kinetics [23]. For example, if a chimera channelrhodopsin consists of the first to fifth transmembrane helixes from ChR2 and the sixth to seventh helixes from ChR1, the photocurrent was enhanced in whole-cell conductance. On the other hand, by further replacement of the sixth transmembrane domain (“*f*”-to-“*F*” replacement) reduced the effective conductance to as small as that of ChR1. It is thus predicted that the domain “*F/f*” is involved in the conductance; high with “*f*” and low with “*F*”. In the present study we did the reverse experiment, that is, replaced “*F*” of ChR1 with its counterpart from ChR2, and produced a chimera, ChR1-*f*. When expressed in HEK293 cells, this replacement enhanced the effective conductance without changing the reversal potential and the action spectrum. It is, thus, suggested that the “*F/f*” segment is one of the molecular determinants of photocurrent conductance by either regulating the expression of the molecule to the plasma membrane or regulating the ion flux per a molecule.

The seventh transmembrane domain has been suggested to be involved in the color tuning of channelrhodopsins [23]. Actually, the “G”-to-“g” replacement definitely blue-shifted the action spectrum of ChR1. This was also the same in the present study using ChR1-*f*. We further subdivided the “*G/g*” domain into two subdomains; the N-terminal 20 amino acids before the retinal-binding Lys ( “*G*_1_/*g*_1_”) and the C-terminal 49 amino acids after it (“*G*_2_/*g*_2_”). We found the “*G*_1_”-to-“*g*_1_” replacement blue-shifted the action spectrum, whereas the “*G*_2_”-to-“*g*_2_” replacement had no effect. These results clearly suggest that the “*G*_1_/*g*_1_” subdomain is one of the molecular determinants involved in the wavelength sensitivity.

When expressed in HEK293 cells, the photocurrent amplitude of ChR1-*fg*_2_ (ChRGR) was relatively large in response to the green light if compared to that of ChR2 or ChR2-H134R (Figure S1). Therefore, it is the first practical optogenetic molecule with the ChR1 backbone. It was also preferentially sensitive to green light as one of the *Volvox*-derived channelrhodopsins (VChR1). Its average photocurrent evoked by 520 nm light was even larger than that of VChR1 probably because of its efficient membrane expression. The ON and OFF kinetics of ChRGR photocurrent was as fast as other ChR variants with fast photocurrent kinetics [23], [32] and even faster than ChR2 or ChR2-H134R [9], [14], [22] (Figure S6). Remarkably, the ChRGR photocurrent was small in the desensitization and was even smaller than ChR1. It was also rapid in the recovery from desensitization. Its recovery time constant was 1.3 s at room temperature (27–30°C), which was smaller than the corresponding value of ChR1, ChR2 or ChR2-H134R (3–20 s) (Figure S6) [14], [21], [22], [33]. These kinetic properties of ChRGR appeared to be optimal for optogenetic injection of the patterned current into a neuron (opto-current clamp). Similar to ChR1 [21], the ChRGR was sensitive to pH (Figure S7). The reversal potential was pH-dependent and was positively shifted with the reduction of pH. The τ_OFF_ was also pH-dependent although τ_ON_ and desensitization were not. Therefore, the fast kinetics of ChRGR appears to be optimized at pH 7.4.

In our experiments with the L5 pyramidal neurons the ChRGR molecules were not detectable in the spines within twelve hours after transfection although they distributed to the distal end of tufted dendrites. It is possible that it would take additional hours for the molecules to distribute in spines. Alternatively, the molecules might have some regional preferences. In either case it may be, the green light should depolarized the distal dendritic region to a certain level which is a function of the regional density of the ChRGR, the light power density at the region and the local input resistance. Since ChRGR was also distributed in the axonal membrane, the effects of simultaneous depolarization of a region including soma, dendrites and axon were investigated by the opto-current clamp of L5 pyramidal neurons. Our results suggested that an individual neuron has its unique firing frequency where the probability of action potential generation is expected to be highest [34]. This frequency was 3–10 Hz when a square light pulse was applied. The robust firing time window of RSSL also corresponded to this frequency band.

**Figure 7:**
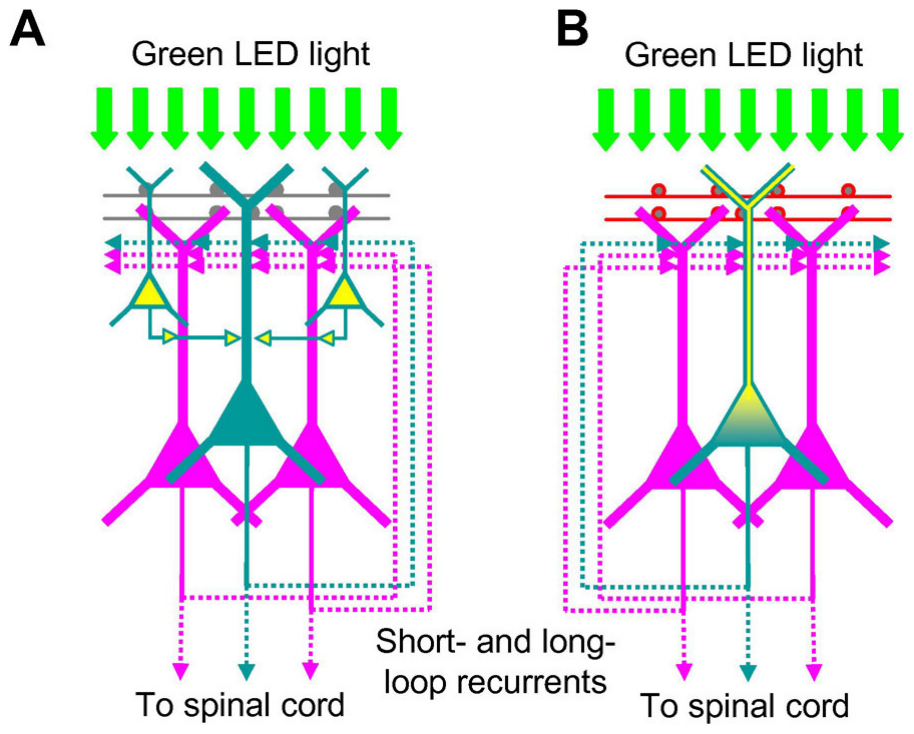
Two hypotheses on the opto-current-clamp actuation of the cortical circuits. **A**. The green LED light may directly fire the neurons in the surface layers (yellow), but not those in the deep layers (dark green). **B**. The green LED light may directly drive the ChRGR-expressing L5 pyramidal neuron with the intrinsic synaptic bombardments (red boutons) on the tufted apical dendrites.

When the motor cortex was exposed to the light, it was expected that the neurons in the superficial layers would be depolarized to fire directly (Figure 7A). This is consistent with our finding that some of the ChRGR-expressing neurons in these layers were also immunoreactive to anti-c-Fos. Although these neurons would fire asynchronously during RSSL and consist of both excitatory and inhibitory traits [35], [36], their transmissions would be integrated in the major output neurons [37]. It was also expected that light would depolarize directly the tufted apical dendrites of a ChRGR-expressing L5 pyramidal neuron (Figure 7B). The probability of evoking action potentials would be enhanced during the depolarizing phase of the light-evoked membrane oscillation because of the asynchronous bombardments from intrinsic synaptic inputs [38]. Light-evoked depolarization also facilitates the dendrite to evoke action potentials that are dependent on either Ca^2+^ channels or NMDA receptors [39]. Consistent with this notion, some of the ChRGR-expressing L5 pyramidal neurons were also immunoreactive to anti-c-Fos. It is possible that above two responses in concern enhanced the LFP response at a preferential frequency of 3–10 Hz. Long-range as well as local recurrent circuits also appear to be involved since the LFP response does not always coincide with the light illumination [40], [41]. The spread of activity was evidenced by the fact that there were many c-Fos neurons that were negative for ChRGR in a region of the motor cortex. Some of them are suggested to project to spinal cord motor neurons directly or indirectly [42], [43] as the light-evoked cortical responses were accompanied by enhanced EMG responses.

The LFP power was indeed small at any frequency band under anesthesia. Therefore, the local network appears to be in a stable basin state where the afferent inputs, if any, hardly shift it to the activated semistable state [44]. Our opto-current-clamp study suggests that the depolarization of a small number of neurons wakes up the motor cortical network over some critical point to the activated state where the positive feedback implemented by the recurrent connections maintains a high probability of firing and generates the efferent output to the spinal cord [45].

## Materials and Methods

### Ethics Statement

All animal experiments were approved by the Tohoku University Committee for Animal Experiments (Approval No. 21LsA-44) and were carried out in accordance with the Guidelines for Animal Experiment and Related Activities in Tohoku University as well as the guiding principles of the Physiological Society of Japan and the NIH.

### Plasmid Construction and Expression

Chimeric channelopsins (chops) between chop1 (amino acids 1–345; accession number, AB058890/AF385748, a generous gift from Dr. T. Takahashi, Toho University, Japan) and chop2(1–315) (accession number, AB058891/AF461397, a generous gift from Dr. G. Nagel, Universität Würzburg, Germany) with 5′-EcoRI and 3′-BamHI restriction sites were constructed by overlap extension PCR as described previously [23] using KOD plus DNA polymerase (Toyobo, Osaka, Japan). A chimeric chop fragment was obtained, purified, digested by EcoRI and BamHI and subcloned in-frame into the plasmid pVenus-N1 which has the Venus construct (a generous gift from Dr. A. Miyawaki, RIKEN BSI, Japan) [22]. Coding regions in all constructed plasmids were fully sequenced to verify that no undesired mutations had been introduced by PCR. HEK293 cells were cultured at 37°C and 5% CO_2_ in D-MEM (Wako, Osaka, Japan) supplemented with 10% fetal bovine serum and transfected using Effectene Transfection Reagent (Qiagen, Tokyo, Japan) according to the manufacturer's instructions. Twenty-four hours post transfection, the cells were replated onto the collagen-coated glass coverslips for the electrophysiology. We did not supplement the culture and experimental media with retinal, but observed enough large photocurrents for the following experiments.

### Evaluation of photocurrents

HEK cells were prepared for electrophysiological recordings 48 hours after transfection. Fluorescence-labeled isolated cells were identified under conventional epi-fluorescence microscopy (BH2-RFC, Olympus, Tokyo, Japan) equipped with a 60× water-immersion objective (LUMplanPl/IR60x, Olympus). The photocurrents were recorded under the whole-cell patch clamp of a conventional system consisting of an amplifier (EPC 8, HEKA Elektronik Dr. Schulze GmbH, Germany) and A/D converter (Digidata 1200B, Molecular Devices Co., Sunnyvale, CA) The standard patch pipette solution contained (in mM), 120 CsOH, 100 glutamate, 50 HEPES, 2.5 MgCl, 2.5 MgATP, 5 Na_2_EGTA, 1.2 leupeptin (Sigma) and pH 7.2, adjusted by 1N CsOH. The access resistance was 10–20 MΩ and was monitored throughout the recording. The cells were continuously superfused (1–2 ml/min) by standard Tyrode solution (in mM:138 NaCl, 3 KCl, 1 CaCl_2_, 1 MgCl_2_, 10 HEPES, 4 NaOH, 11 glucose, pH 7.4 by 1N HCl). Most of the experiments were performed at room temperature (27–30°C) unless otherwise noted. For studying the I–V relationship, Cs in the patch pipette solution was replaced with Na, and the liquid junctional potentials of −8 mV were corrected for. For the wavelength-response relationship monochromatic light at 400–560 nm (width, 10 nm) of 1 s duration was applied every 20 s under a conventional epifluorescence system equipped with a Xenon lamp and electromagnetic shutter (CAM-230, JASCO, Tokyo, Japan). Usually the photocurrent was measured by 2–3 repeats of a protocol, in which the wavelength was changed in the order of 460, 480, 500, 520, 540, 560, 560, 540, 520, 500, 480, 460, 440, 420, 400, 400, 420, 440 and 460, and was averaged for each wavelength. The light power density at each wavelength was directly measured by a thermopile (MIR-100Q, Mitsubishi Oil Chemicals, Tokyo, Japan), and was (in mWmm^−2^) 0.021 (400 nm), 0.018 (420 nm), 0.027 (440 nm), 0.027 (460 nm), 0.018 (480 nm), 0.021 (500 nm), 0.015 (520 nm), 0.014 (540 nm) or 0.012 (560 nm), respectively. To investigate the action spectrum, the amplitude of the peak photocurrent at each wavelength was divided by the light power density, multiplied by the wavelength and normalized to the value at 480 nm. For analysis of the photocurrent (*I*)-voltage (*V*) relationship and the photocurrent kinetics we used a green LED (505±15 nm, LXHL-NE98, Lumileds Lighting Inc. San Jose, CA) regulated by a pulse generator (SEN-7203, Nihon Kohden, Tokyo, Japan) and computer software (pCLAMP 9, Molecular Devices Co.). The maximal light power density of LED light was 0.77 mWmm^−2^ at the focus. The effective conductance was calculated for each cell by dividing the slope of the *I–V* relationship between −48 and 32 mV by the input capacitance. The effective conductance was compared as relative to that of ChR1. The turning-on (ON) and -off (OFF) transitions were analyzed by fitting the photocurrent using the simplex method of non-linear least-squares protocol of the appropriate software (Clampfit 9.2 and 10.1 Molecular Devices Co.).

### Sindbis pseudovirions transfection

A fragment of ChRGR-Venus was generated by PCR and subcloned into pSinRep5 (Invitrogen). The recombinant Sindbis pseudovirion was generated according to the manufacturer's instructions. Briefly, RNAs were transcribed from this plasmid and DH(26S) helper DNA using MEGAscript SP6 Kit (Ambion, Austin, TX). BHK cells were electroporated with these RNAs and were grown for 24 hr at 37°C, 5% CO_2_ in α-MEM containing 5% fetal bovine serum before collecting the supernatant. The titer tested on BHK cells, determined after counting the fluorescent cells infected with serial dilution of the virus stocks, was 1.9×10^6^ infectious particles per ml. Recombinant viral stocks were stored at −80°C.

All experiments were done using male C57BL/6J mice (2–3 weeks-old, 7–10 gBW) under anaesthetized by intraperitoneal injection of ketamine-xylazine mixture (50 mg/kgBW ketamine, Daiichi Sankyo Co. Ltd., Tokyo, Japan and 10 mg/kgBW xylazine, Sigma-Aldrich, St. Louis, MO, USA). The viral solution was stereotaxically injected into the primary motor area of the right hemisphere with the following coordinates: anteroposterior from bregma 0.33 mm, lateral from bregma 1.58 mm and ventral 0.5 mm. The body temperature was kept at 37°C by a chemical bedding heater during whole surgery.

### Electrophysiology of L5 pyramidal neurons

Twelve hours after injection of the Sindbis pseudovirion vectors, the mice were ether-anesthetized, killed and the cerebral cortex was quickly removed. Sagittal brain slices 250 µm in thickness were prepared using a vibratome (Leica, VT1000s, Wetzlar, Germany) in ice-cold cutting buffer (in mM: 229 Mannitol, 3 KCl, 26 NaHCO_3_, 1 H_3_PO_4_, 7 MgCl_2_ and 11 glucose) bubbled with mixed gas containing 95% O_2_ and 5% CO_2_. Brain slices were further incubated in artificial cerebrospinal fluid (in mM: 114 NaCl, 2.5 KCl, 26 NaHCO_3_, 1 NaH_2_PO_4_, 10 Mannitol, 1.25 MgCl_2_, 2.5 CaCl_2_ and 11 glucose) bubbled with the above described mixed gas at 34°C for one hour for the recovery. Although high levels of acetylcholine have been suggested to set the L5 pyramidal neurons to the appropriate dynamics in an awake animal [46], [47], the cholinergic tone was absent in the slice. Therefore, it was artificially restored by bath application of the nonhydrolyzable cholinergic agonist carbachol (5 µM, Nacalai Tesque, Kyoto, Japan).

The ChRGR-Venus-expressing layer 5 (L5) pyramidal neurons were visually identified in an acute slice of cerebral cortex under conventional epi-fluorescent microscopy system (BH-2, Olympus Optical Co., Tokyo, Japan) equipped with a 60× water-immersion objective (LUMplanPl/IR60x, Olympus) and a conventional filter cube (excitation, 495 nm; dichroic mirror, 505 nm; barrier filter, 515 nm). Electrophysiological recording was performed at 34±2°C (UTC-1000, Ampere Inc., Tokyo, Japan) under the whole-cell patch clamp from the soma using an amplifier (EPC 8, HEKA Elektronik Dr. Schulze GmbH, Germany). The patch pipette solution was composed of (in mM) 115 potassium gluconate, 20 KCl, 10 HEPES, 2 MgCl_2_, 0.2 Na_2_EGTA, 2.5 MgATP, 0.3 Na_2_GTP and 1.2 leupeptin (Sigma-Aldrich) (pH 7.2 by 1 N KOH). In some experiments, biocytin (2 mg/mL, Sigma-Aldrich) was included for the following morphological studies. The pipette resistance, measured directly, was 3.09±0.30 MΩ (n = 16). The liquid junction potential, directly measured, was −9.0 mV and was compensated for. To illuminate the light on the L5 pyramidal neuron under the patch clamp we used the same green LED system as for HEK293 cells. The oscillatory LED light was generated according to the following voltage change [48]:
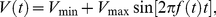
(1)where *V*_min_ and *V*_max_ are the minimal and the maximal voltages driving the LED. The oscillation frequency is swept from the minimal frequency, *f*_min_, to the maximal frequency, *f*_max_, following the equation [27]:
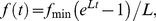
(2)where
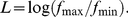
(3)However, since the LED light power density was 0 during *V*<*V*_min_, only a positive range of the above function was employed (the rectified sinusoidal sweep of light, RSSL).

Data were low-pass filtered at 1 kHz, sampled digitally at 2 kHz using an A/D converter (Digidata 1200B, Molecular Devices Co.) with a software (Clampex 9.2, Molecular Devices Co.) and analyzed by other software (Clampfit 10.2, Molecular Devices Co.).

### Local field potentials (LFP) and electromyogram (EMG)

All the experiments were performed 12 hours after viral injection. At first, mice were anesthetized by intraperitoneal injection of ketamine-xylazine mixture (50 mg/kgBW ketamine, Daiichi Sankyo Co. Ltd. and 10 mg/kgBW xylazine, Sigma-Aldrich). The anesthesia was maintained during the recording by adding small amounts of ketamine-xylazine mixture every 30 min. The head of each mouse was fixed on the stage of the microscope (Labophoto, Nikon, Tokyo, Japan) using tooth and ear bars. A small area (diameter, 2 mm) was craniotomized using a dental drill while the dura was kept intact. A recording electrode was made from coated stainless steel wire (0.1 mm), inserted stereotaxically into the injected site at the right hemisphere, while the reference electrode was inserted into the left hemisphere according to stereotaxial coordinates (anteroposterior −0.42 mm from bregma; lateral −1.58 mm from bregma). Both LFP electrodes were fixed on the skull using dental cement (Fuji I, GC Co., Tokyo, Japan). To record the EMG, two coated stainless steel wires with straight bared ends (0.1 mm) were directly inserted into extensor muscles of the left forelimb, triceps brachii and carpi radialis brevis, respectively. Another wire was inserted into the triceps brachii muscle of the right forelimb as control. A green LED (505±15 nm, LXHL-NE98, Lumileds Lighting Inc.) was driven by a custom-made booster and computer software (pCLAMP 9, Molecular Devices Co.) and focused on the dura membrane through a microscope objective (WPlan 10, Olympus). The maximal LED light power density was 1.12 mWmm^−2^ on the focal plane. Both LFP and EMG signals were band-pass-filtered (1–300 Hz) by a custom-made amplifier (×100,000) sampled at 1 kHz and stored in the computer by software (Clampex 9, Molecular Devices Co.). The power spectra of LFP or EMG were extracted from 10 repetitive traces using Clampfit 10.2 software and averaged for the statistical analysis.

We analyzed the time-frequency energy distribution of the stimulation-evoked response by continuous wavelet transformation [49]. We used the following equation:
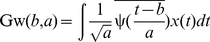
(4)

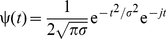
(5)where x(t) is the evoked response in time (*t*) domain, Ψ(*t*) is the mother wavelet, a and b are the scaling factor and translation, respectively. For the mother wavelet, we used the Gabor function in equation (2) with σ being the central frequency of 2 Hz. We varied the scaling factor *a* to explore frequencies ranging from 1 to 100 Hz. The above equation (1) yielded a series of coefficients that represent the temporal evolution of the frequency content, which were plotted on a pseudocolored 2D graph.

### Immunohistochemistry

To investigate the morphological features of the recorded neurons, the cortical slices were immediately fixed after a series of experiments with 4% paraformaldehyde (PFA) in PBS (0.1 M, pH 7.4) for 30 min at 4°C and then washed in PBS. After replacing with 30% sucrose (w/v), each of them was further sliced into 20 µm-thick sections using a freezing microtome (CM 3050S, Leica). The slices were blocked in PBS including 5% normal donkey serum, 0.1% Triton X-100 and 0.25% λ-carrageenan overnight, then treated with the following primary antibodies in PBS including 5% donkey serum and 0.1% Triton X-100. They were reacted with rabbit anti-EGFP IgG (1∶1,000; a generous gift from Drs T. Kaneko and K. Nakamura, Kyoto University, Japan) at 4°C for 24 hours and then with anti rabbit IgG (Alexa Fluor 488 conjugate, 1∶500, Molecular Probes, Oregon, USA) and streptavidin (Alexa Fluor 546 conjugate, 1∶500, Molecular Probes, Oregon USA ) at room temperature for 5–6 hours. Finally the slices were mounted with Permafluor (Immunotech, Marseille, France) and coverslipped.

To investigate the c-Fos expression, the anesthetized mice were transcardially perfused with 0.01M PBS, then with 4% paraformaldehyde (PFA) in PBS immediately after the above electrophysiological recordings. The pre-fixed brains were removed, and post-fixed overnight in 4% PFA at 4°C. For cryoprotection, the brains were kept overnight in a PBS solution each with graded concentrations of sucrose from 10 to 30% (w/v). The slices were cut into 16-µm-thick coronal sections on a freezing microtome (CM 3050S, Leica). The sections were washed in 0.025 M PBS followed by blocking buffer (2.5% normal goat serum, 0.1% Triton X-100 and 0.25% λ-carrageenan in PBS), incubated with anti-c-Fos rabbit antibody (Ab-5, 1∶1,000, Calbiochem-Merk KGaA, Darmstadt, Germany) and anti-GFP rat antibody (1∶500, Nacalai Tesque Inc. Kyoto, Japan) for 48 hours at 4°C. Then, the sections were incubated for 3 hours with biotinylated anti-rabbit-IgG goat antibody (1∶200, Jackson, PA, USA). Finally, the slices were treated with streptavidin (Alexa Fluor 546 conjugate, 1∶200, Molecular Probes) and anti-rat IgG (Alexa Fluor 488 conjugate, 1∶200, Molecular Probes) for fluorescence visualization. The incubation was carried out at room temperature in the blocking buffer, followed by a rinse with PBS. Thereafter, the slices were mounted with Permafluor (Immunotech) and coverslipped.

Each specimen was analyzed three-dimensionally with a z-axis interval of 0.60–0.64 µm under conventional confocal laser-microscopy (LSM510META, Carl Zeiss, Oberkochen, Germany) equipped with a 40× objective unless otherwise noted. The images were corrected for brightness and contrast using conventional software (LSM Image Browser and ImageJ, http://rsb.info.nih.gov/ij/).

### Statistics

All data in the text and figures are presented as means ± S.E.M. (number of observations). Statistical significance was tested by the Wilcoxon signed-ranks test for paired data and by the Mann–Whitney *U*-test for unpaired data. The significance limit was set at P = 0.05 in all tests.

## Supporting Information

Table S1 Basic electrophysiological parameters of L5 pyramidal neurons.
(0.04 MB PDF)

Figure S1 Photocurrent amplitude of ChRGR. ChRGR (ChR1-*fg2*) was expressed in a HEK cell and its photocurrent was measured at each wavelength. Here, the ChRGR photocurrent (green diamond) was compared with the corresponding photocurrent of either ChR2 (blue circle), ChR2-H134R (light blue triangle), or VChR1 (yellow square). A, C, E. the peak currents (I_peak_). B, D, F. the steady-state photocurrents (I_ss_). Each symbol and bars are mean and SEM of data, n = 8 (ChRGR), n = 8 (ChR2), n = 9 (ChR2-H134R) and n = 6 (VChR1). * indicates that the difference was significant (P<0.05, Mann-Whitney *U*-test). The light power density at each wavelength was (in mWmm^−2^) 0.021 (400 nm), 0.018 (420 nm), 0.027 (440 nm), 0.027 (460 nm), 0.018 (480 nm), 0.021 (500 nm), 0.015 (520 nm), 0.014 (540 nm) or 0.012 (560 nm), respectively. It was relatively small at 480 nm and over 520 nm because of the spectral properties of the light source (Xenon arc lamp).
(0.06 MB PDF)

Figure S2 Membrane properties of ChRGR-Venus-expressing neurons. A. Membrane potential responses (bottom traces) to current injections from the patch electrode (top). B. Frequency of action potentials as a function of injected current; ChRGR-Venus-expressing neurons and non-expressing controls. C. Typical current-clamp responses of a L5 pyramidal neuron to the green LED pulses. The external solution included 1 µM TTX to suppress the generation of action potentials. The resting potential was −73 mV and the membrane time constant was 104 ms.
(0.06 MB PDF)

Figure S3 Frequency responsiveness of neuronal membrane potential. The rectified sinusoidal sweep of light (RSSL) from 0.1 to 100 Hz (top green), the photocurrent response of an L5 pyramidal neuron under voltage clamp (*I*, brown), and the membrane potential (*V*, blue) from the same neuron. To suppress the generation of action potentials, TTX (1 µM) was included in the external solution. Note that the membrane potential change became small with the increase of frequency because of the large membrane time constant of this neuron (33 ms).
(0.05 MB PDF)

Figure S4 Non-linear photocurrent responses. The peak (magenta diamond) and steady-state photocurrent amplitude (closed circle) as functions of green LED power density. The lines were drawn fitting to Michaelis-Menten relationship: peak (K_D_, 0.10 mWmm^−2^; I_max_, 0.19 nA) and steady-state (K_D_, 0.10 mWmm^−2^; I_max_, 0.17 nA). Recorded from ChRGR-expressing L5 pyramidal neurons (mean ± SEM, n = 4).
(0.04 MB PDF)

Figure S5 Distribution of ChRGR-expressing neurons. (Left) The number of neurons expressing ChRGR-Venus was estimated for each depth. The ChRGR-expressing neurons which also expressed c-Fos at higher levels were counted as indicated in white columns. (Right) The LFP (black lines) and EMG (red lines) recorded from each animal. The data from mouse #2 was typical and used in the text-figure 3. Methods: The post-fixed mouse brain was sliced into 16-mm-thick coronal sections on a freezing microtome (CM 3050S, Leica) and immunohistochemically labeled with anti-EGFP and anti-c-Fos. The numbers of fluorescent neurons were counted under confocal microscopy (LSM510META, Oberkochen, Germany) using one of every 5 slices (80 µm). Total numbers of neurons were estimated for each depth window by linear estimation. As for anti-c-Fos, the intensity of fluorescence was variable from one cell to another. The cell expressing c-Fos at a higher level was visually identified relative to the background fluorescence.
(0.32 MB PDF)

Figure S6 Photocurrent kinetics of ChR2-H134R. A–C. ChR2-H134R was expressed in a HEK cell and its photocurrent was evoked by 1s pulse of green LED of various strenth (light blue triangle, mean and SEM). (A) The apparent ON time constant (τ_ON_)-light power density relationships. (B) The effective OFF time constant (τ_OFF_)-light power density relationships. (C) The desensitization-light power density relationships. D. The recovery time course of the desensitizing component. The process was fitted to a single exponential relationship (time constant, 3 s). Each green line is the relationship obtained from ChRGR that is copied from text-Figure 2D–F or 2J.
(0.05 MB PDF)

Figure S7 Sensitivity to pH. A and B. ChRGR (ChR1-*fg2*) was expressed in a HEK cell and its photocurrent was measured at each membrane potential of −48 (blue), −28 (magenta), −8 (brown), 12 (green) or 32 mV (purple). The pH of external solution was adjusted to 7.4 (A) or 6.0 (B). C and D. The peak currents (I_peak_, open diamond) and the steady-state photocurrents (I_ss_, closed circle) as functions of membrane potential (*V*). pH 7.4 (C) and 6.0 (D). E. The pH sensitivity of τ_OFF_. The difference was significant (P<0.01, Mann-Whitney *U*-test). F. The pH sensitivity of τ_ON_. G. The pH sensitivity of desensitization.
(0.05 MB PDF)
