# Supplementary material for: Opto-Current-Clamp Actuation of Cortical Neurons Using a Strategically Designed Channelrhodopsin
Source: PLoS One. 2010 Sep 23;5(9):e12893. doi: 10.1371/journal.pone.0012893 (PMC2944835; doi:10.1371/journal.pone.0012893)
Supplement: Table S1 — Basic electrophysiological parameters of L5 pyramidal neurons. (0.04 MB PDF) [file pone.0012893.s001.pdf]

Table S1

Table S1. Basic electrophysiological parameters of L5 pyramidal neurons.

	Control neurons (n = 8)	ChRGR-exprssing neurons (n = 9)
Resting potential (mV)	-61 ± 0.65	-60 ± 1.2
Input resistance (Mohm)	170 ± 16	220 ± 32
Input capacitance (pF)	160 ± 12	170 ± 11
Pipette resistance (Mohm)	3.5 ± 0.06	3.2 ± 0.1
Access resistance (Mohm)	26 ± 4.0	17 ± 1.5

Each number represents mean ± SEM. For each parameter no significance was detected between two groups (P > 0.1, Mann-Whitney U-test).
