# Supplementary material for: Opto-Current-Clamp Actuation of Cortical Neurons Using a Strategically Designed Channelrhodopsin
Source: PLoS One. 2010 Sep 23;5(9):e12893. doi: 10.1371/journal.pone.0012893 (PMC2944835; doi:10.1371/journal.pone.0012893)
Supplement: Figure S1 — Photocurrent amplitude of ChRGR. ChRGR (ChR1-fg2) was expressed in a HEK cell and its photocurrent was measured at each wavelength. Here, the ChRGR photocurrent (green diamond) was compared with the corresponding photocurrent of either ChR2 (blue circle), ChR2-H134R (light blue triangle), or VChR1 (yellow square). A, C, E. the peak currents (Ipeak). B, D, F. the steady-state photocurrents (Iss). Each symbol and bars are mean and SEM of data, n = 8 (ChRGR), n = 8 (ChR2), n = 9 (ChR2-H134R) and n = 6 (VChR1). * indicates that the difference was significant (P<0.05, Mann-Whitney U-test). The light power density at each wavelength was (in mWmm−2) 0.021 (400 nm), 0.018 (420 nm), 0.027 (440 nm), 0.027 (460 nm), 0.018 (480 nm), 0.021 (500 nm), 0.015 (520 nm), 0.014 (540 nm) or 0.012 (560 nm), respectively. It was relatively small at 480 nm and over 520 nm because of the spectral properties of the light source (Xenon arc lamp). (0.06 MB PDF) [file pone.0012893.s002.pdf]

Figure S1

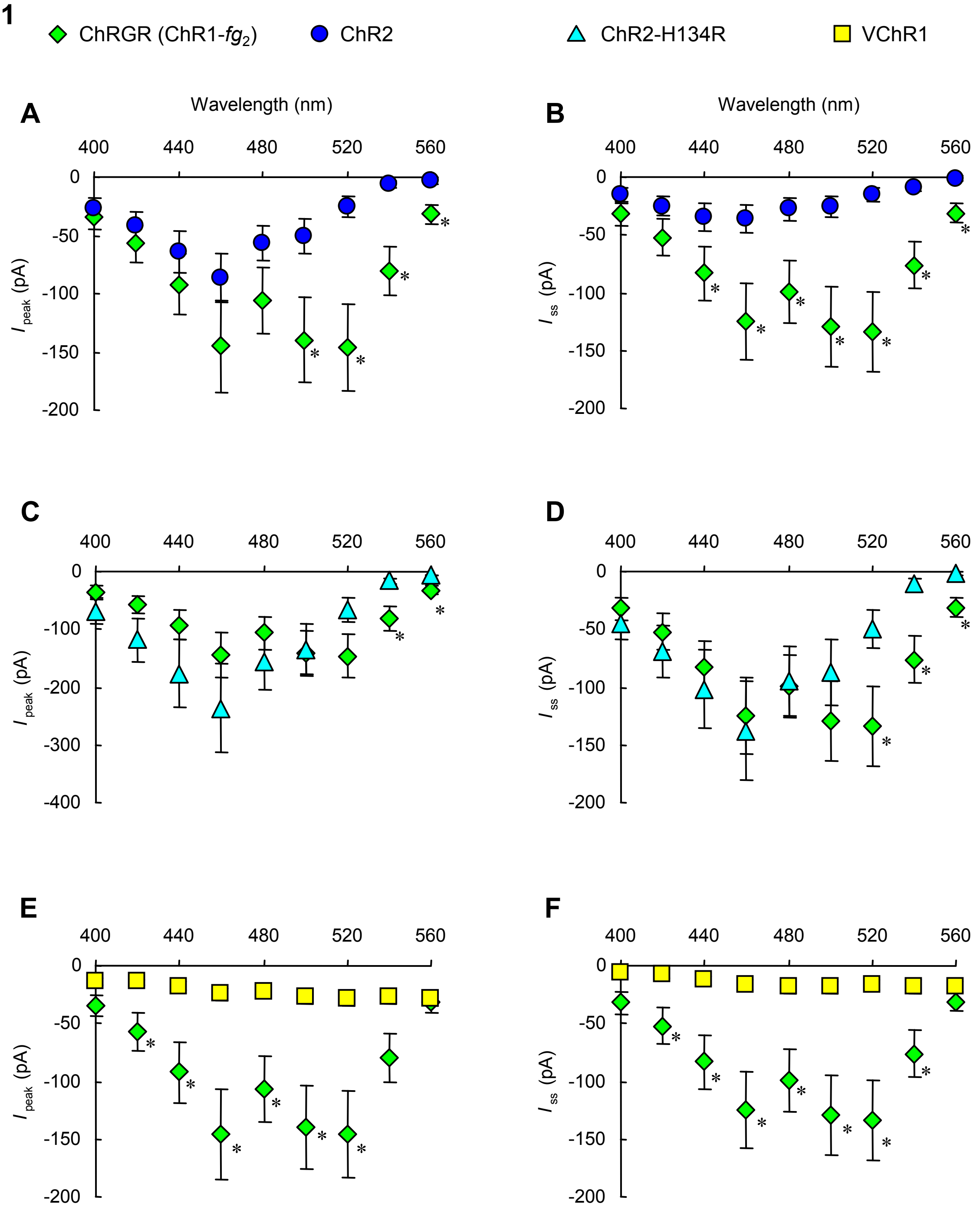

Figure S1. Photocurrent amplitude of ChRGR. ChRGR (ChR1-*fg*$_2$) was expressed in a HEK cell and its photocurrent was measured at each wavelength. Here, the ChRGR photocurrent (green diamond) was compared with the corresponding photocurrent of either ChR2 (blue circle), ChR2-H134R (light blue triangle), or VChR1 (yellow square). **A**, **C**, **E**. The peak currents (I_{peak}). **B**, **D**, **F**. The steady-state photocurrents (I_{ss}). Each symbol and bars are mean and SEM of data, n = 8 (ChRGR), n = 8 (ChR2), n = 9 (ChR2-H134R) and n =6 (VChR1). * indicates that the difference was significant ($P < 0.05$, Mann-Whitney *U*-test). The light power density at each wavelength was (in mWmm-2) 0.021 (400 nm), 0.018 (420 nm), 0.027 (440 nm), 0.027 (460 nm), 0.018 (480 nm), 0.021 (500 nm), 0.015 (520 nm), 0.014 (540 nm) or 0.012 (560 nm), respectively. It was relatively small at 480 nm and over 520 nm because of the spectral properties of the light source (Xenon arc lamp).
