# Supplementary material for: Opto-Current-Clamp Actuation of Cortical Neurons Using a Strategically Designed Channelrhodopsin
Source: PLoS One. 2010 Sep 23;5(9):e12893. doi: 10.1371/journal.pone.0012893 (PMC2944835; doi:10.1371/journal.pone.0012893)
Supplement: Figure S2 — Membrane properties of ChRGR-Venus-expressing neurons. A. Membrane potential responses (bottom traces) to current injections from the patch electrode (top). B. Frequency of action potentials as a function of injected current; ChRGR-Venus-expressing neurons and non-expressing controls. C. Typical current-clamp responses of a L5 pyramidal neuron to the green LED pulses. The external solution included 1 µM TTX to suppress the generation of action potentials. The resting potential was −73 mV and the membrane time constant was 104 ms. (0.06 MB PDF) [file pone.0012893.s003.pdf]

Figure S2

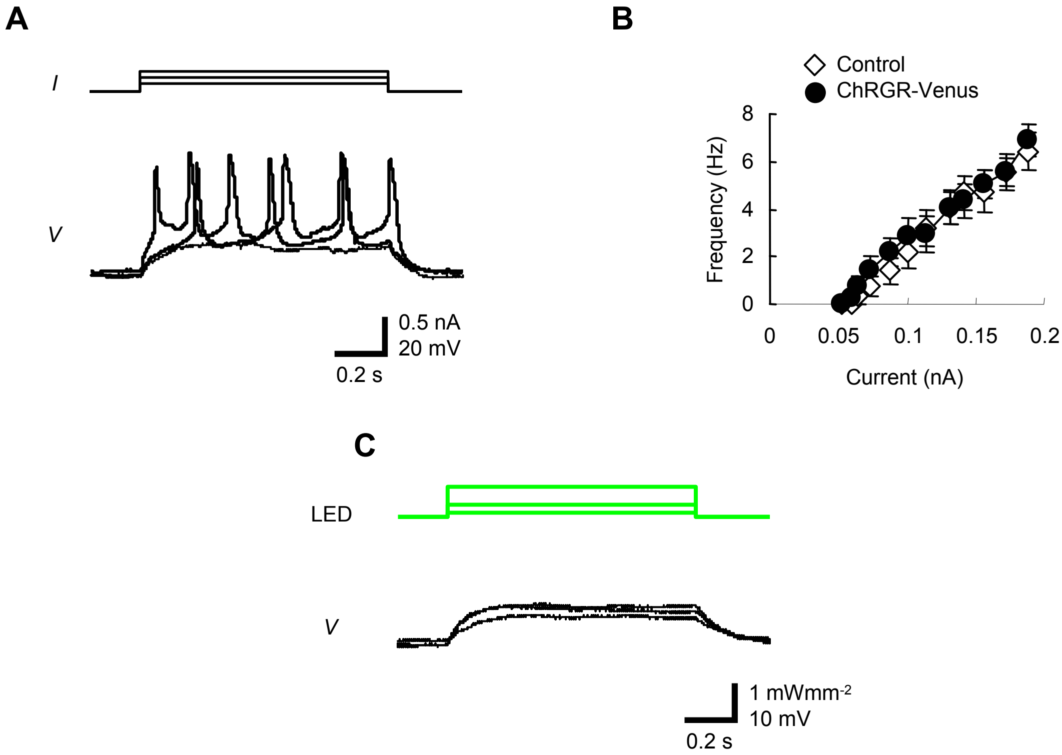

Figure S2. Membrane properties of ChRGR-Venus-expressing neurons. A. Membrane potential responses (*V*, bottom traces) to current injections from the patch electrode (*I*, top). **B.** Frequency of action potentials as a function of injected current; ChRGR-Venus-expressing neurons and non-expressing controls. **C.** Typical current-clamp responses (*V*, bottom traces) of a L5 pyramidal neuron to the green LED pulses (top). The external solution included 1 μM TTX to suppress the generation of action potentials. The relationship between the light power density and the voltage change was non-linear because of the non-linear light power density-photocurrent relationship. The resting potential was -73 mV and the membrane time constant was 104 ms.
