# Supplementary material for: Opto-Current-Clamp Actuation of Cortical Neurons Using a Strategically Designed Channelrhodopsin
Source: PLoS One. 2010 Sep 23;5(9):e12893. doi: 10.1371/journal.pone.0012893 (PMC2944835; doi:10.1371/journal.pone.0012893)
Supplement: Figure S3 — Frequency responsiveness of neuronal membrane potential. The rectified sinusoidal sweep of light (RSSL) from 0.1 to 100 Hz (top green), the photocurrent response of an L5 pyramidal neuron under voltage clamp (I, brown), and the membrane potential (V, blue) from the same neuron. To suppress the generation of action potentials, TTX (1 µM) was included in the external solution. Note that the membrane potential change became small with the increase of frequency because of the large membrane time constant of this neuron (33 ms). (0.05 MB PDF) [file pone.0012893.s004.pdf]

Figure S3

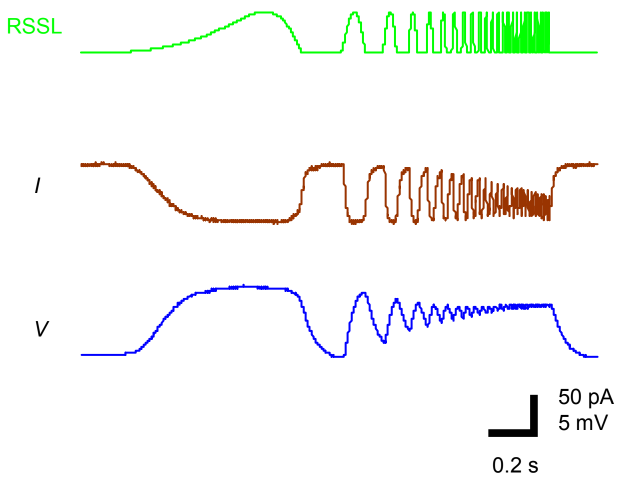

Figure S3. Frequency responsiveness of neuronal membrane potential. The rectified sinusoidal sweep of light (RSSL) from 0.1 to 100 Hz (top green), the photocurrent response of an L5 pyramidal neuron under voltage clamp (*I*, brown), and the membrane potential (*V*, blue) from the same neuron. To suppress the generation of action potentials, TTX (1 μM) was included in the external solution. Note that the membrane potential change became small with the increase of frequency because of the large membrane time constant of this neuron (33 ms).
