# Supplementary material for: Opto-Current-Clamp Actuation of Cortical Neurons Using a Strategically Designed Channelrhodopsin
Source: PLoS One. 2010 Sep 23;5(9):e12893. doi: 10.1371/journal.pone.0012893 (PMC2944835; doi:10.1371/journal.pone.0012893)
Supplement: Figure S4 — Non-linear photocurrent responses. The peak (magenta diamond) and steady-state photocurrent amplitude (closed circle) as functions of green LED power density. The lines were drawn fitting to Michaelis-Menten relationship: peak (KD, 0.10 mWmm−2; Imax, 0.19 nA) and steady-state (KD, 0.10 mWmm−2; Imax, 0.17 nA). Recorded from ChRGR-expressing L5 pyramidal neurons (mean ± SEM, n = 4). (0.04 MB PDF) [file pone.0012893.s005.pdf]

Figure S4

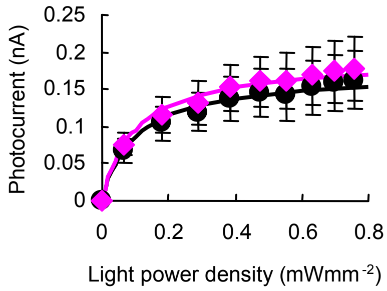

Figure S4. Non-linear photocurrent responses. The peak (magenta diamond) and steady-state photocurrent amplitude (closed circle) as functions of green LED power density. The lines were drawn fitting to Michaelis-Menten relationship: peak (K_D, 0.10 $mWmm^{-2}$; I_{max}, 0.19 nA) and steady-state (K_D, 0.10 $mWmm^{-2}$; I_{max}, 0.17 nA). Recorded from ChRGR-expressing L5 pyramidal neurons (mean ± SEM, n = 4).
