# Supplementary material for: Opto-Current-Clamp Actuation of Cortical Neurons Using a Strategically Designed Channelrhodopsin
Source: PLoS One. 2010 Sep 23;5(9):e12893. doi: 10.1371/journal.pone.0012893 (PMC2944835; doi:10.1371/journal.pone.0012893)
Supplement: Figure S5 — Distribution of ChRGR-expressing neurons. (Left) The number of neurons expressing ChRGR-Venus was estimated for each depth. The ChRGR-expressing neurons which also expressed c-Fos at higher levels were counted as indicated in white columns. (Right) The LFP (black lines) and EMG (red lines) recorded from each animal. The data from mouse #2 was typical and used in the text-figure 3. Methods: The post-fixed mouse brain was sliced into 16-mm-thick coronal sections on a freezing microtome (CM 3050S, Leica) and immunohistochemically labeled with anti-EGFP and anti-c-Fos. The numbers of fluorescent neurons were counted under confocal microscopy (LSM510META, Oberkochen, Germany) using one of every 5 slices (80 µm). Total numbers of neurons were estimated for each depth window by linear estimation. As for anti-c-Fos, the intensity of fluorescence was variable from one cell to another. The cell expressing c-Fos at a higher level was visually identified relative to the background fluorescence. (0.32 MB PDF) [file pone.0012893.s006.pdf]

Figure S5

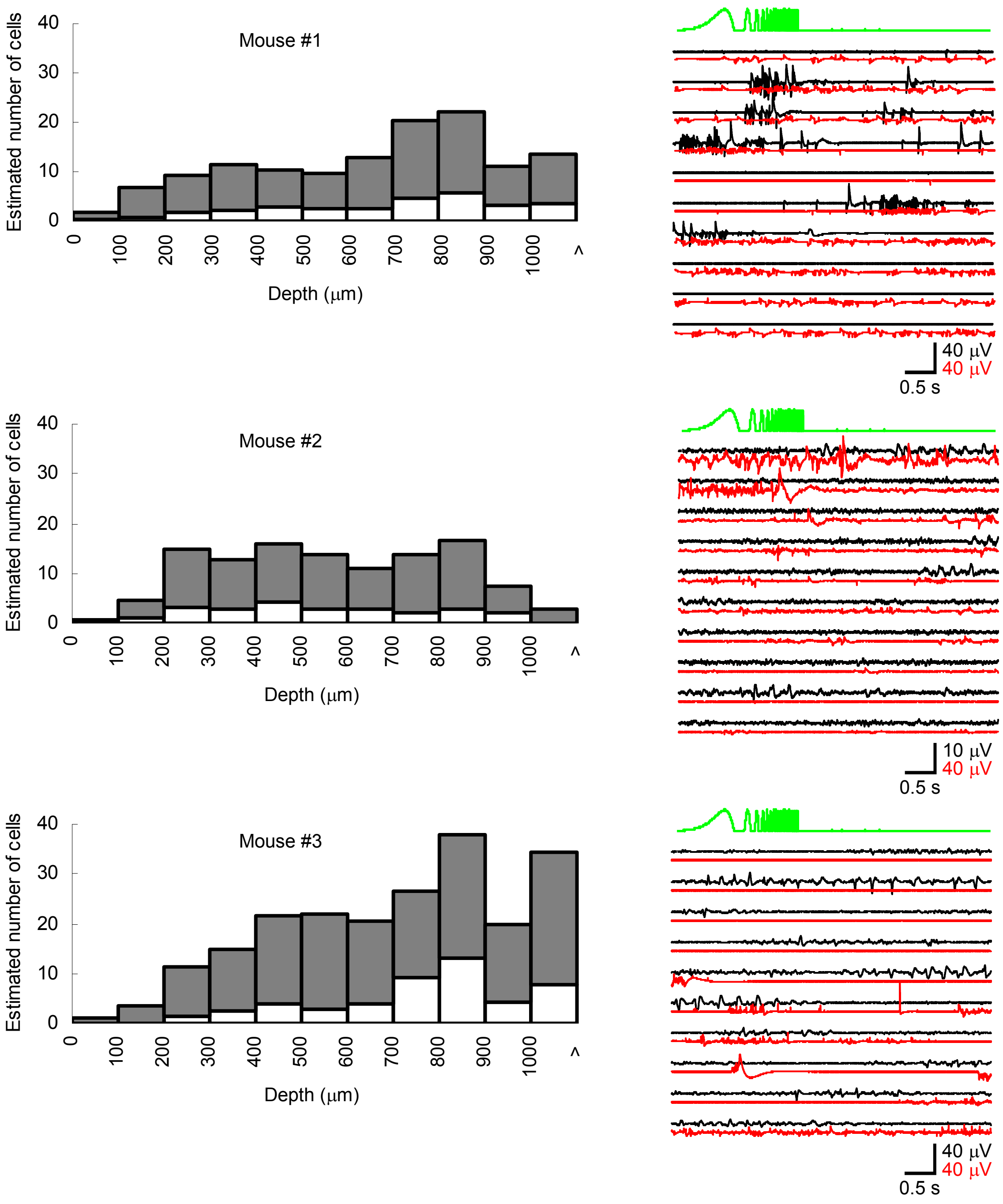

Figure S5. Distribution of ChRGR-expressing neurons. (Left) The number of neurons expressing ChRGR-Venus was estimated for each depth. The ChRGR-expressing neurons which also expressed c-Fos at higher levels were counted as indicated in white columns. (Right) The LFP (black lines) and EMG (red lines) recorded from each animal. The data from mouse #2 was typical and used in the text-figure 3.

<u>Methods:</u>

The post-fixed mouse brain was sliced into 16-μm-thick coronal sections on a freezing microtome (CM 3050S, Leica) and immunohistochemically labeled with anti-EGFP and anti-c-Fos. The numbers of fluorescent neurons were counted under confocal microscopy (LSM510META, Oberkochen, Germany) using one of every 5 slices (80 μm). Total numbers of neurons were estimated for each depth window by linear estimation. As for anti-c-Fos, the intensity of fluorescence was variable from one cell to another. The cell expressing c-Fos at a higher level was visually identified relative to the background fluorescence.
