# Supplementary material for: Opto-Current-Clamp Actuation of Cortical Neurons Using a Strategically Designed Channelrhodopsin
Source: PLoS One. 2010 Sep 23;5(9):e12893. doi: 10.1371/journal.pone.0012893 (PMC2944835; doi:10.1371/journal.pone.0012893)
Supplement: Figure S6 — Photocurrent kinetics of ChR2-H134R. A–C. ChR2-H134R was expressed in a HEK cell and its photocurrent was evoked by 1s pulse of green LED of various strenth (light blue triangle, mean and SEM). (A) The apparent ON time constant (τON)-light power density relationships. (B) The effective OFF time constant (τOFF)-light power density relationships. (C) The desensitization-light power density relationships. D. The recovery time course of the desensitizing component. The process was fitted to a single exponential relationship (time constant, 3 s). Each green line is the relationship obtained from ChRGR that is copied from text-Figure 2D–F or 2J. (0.05 MB PDF) [file pone.0012893.s007.pdf]

Figure S6

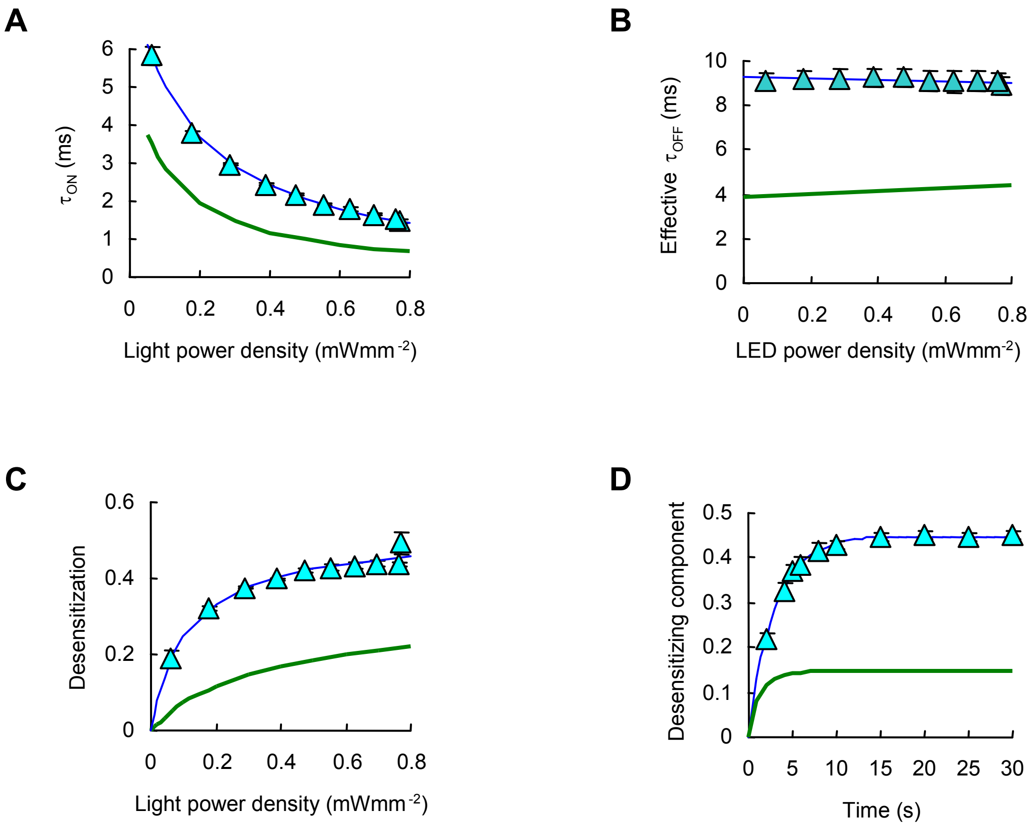

Figure S6. Photocurrent kinetics of ChR2-H134R. A-C. ChR2-H134R was expressed in a HEK cell and its photocurrent was evoked by 1s pulse of green LED of various strenth (light blue triangle, mean and SEM). (A) The apparent ON time constant (τ_{ON})-light power density relationships. (B) The effective OFF time constant (τ_{OFF})-light power density relationships. (C) The desensitization-light power density relationships. **D**. The recovery time course of the desensitizing component. The process was fitted to a single exponential relationship (time constant, 3 s). Each green line is the relationship obtained from ChRGR that is copied from text-Figure 2D-F or 2J.
