# Supplementary material for: Opto-Current-Clamp Actuation of Cortical Neurons Using a Strategically Designed Channelrhodopsin
Source: PLoS One. 2010 Sep 23;5(9):e12893. doi: 10.1371/journal.pone.0012893 (PMC2944835; doi:10.1371/journal.pone.0012893)
Supplement: Figure S7 — Sensitivity to pH. A and B. ChRGR (ChR1-fg2) was expressed in a HEK cell and its photocurrent was measured at each membrane potential of −48 (blue), −28 (magenta), −8 (brown), 12 (green) or 32 mV (purple). The pH of external solution was adjusted to 7.4 (A) or 6.0 (B). C and D. The peak currents (Ipeak, open diamond) and the steady-state photocurrents (Iss, closed circle) as functions of membrane potential (V). pH 7.4 (C) and 6.0 (D). E. The pH sensitivity of τOFF. The difference was significant (P<0.01, Mann-Whitney U-test). F. The pH sensitivity of τON. G. The pH sensitivity of desensitization. (0.05 MB PDF) [file pone.0012893.s008.pdf]

Figure S7

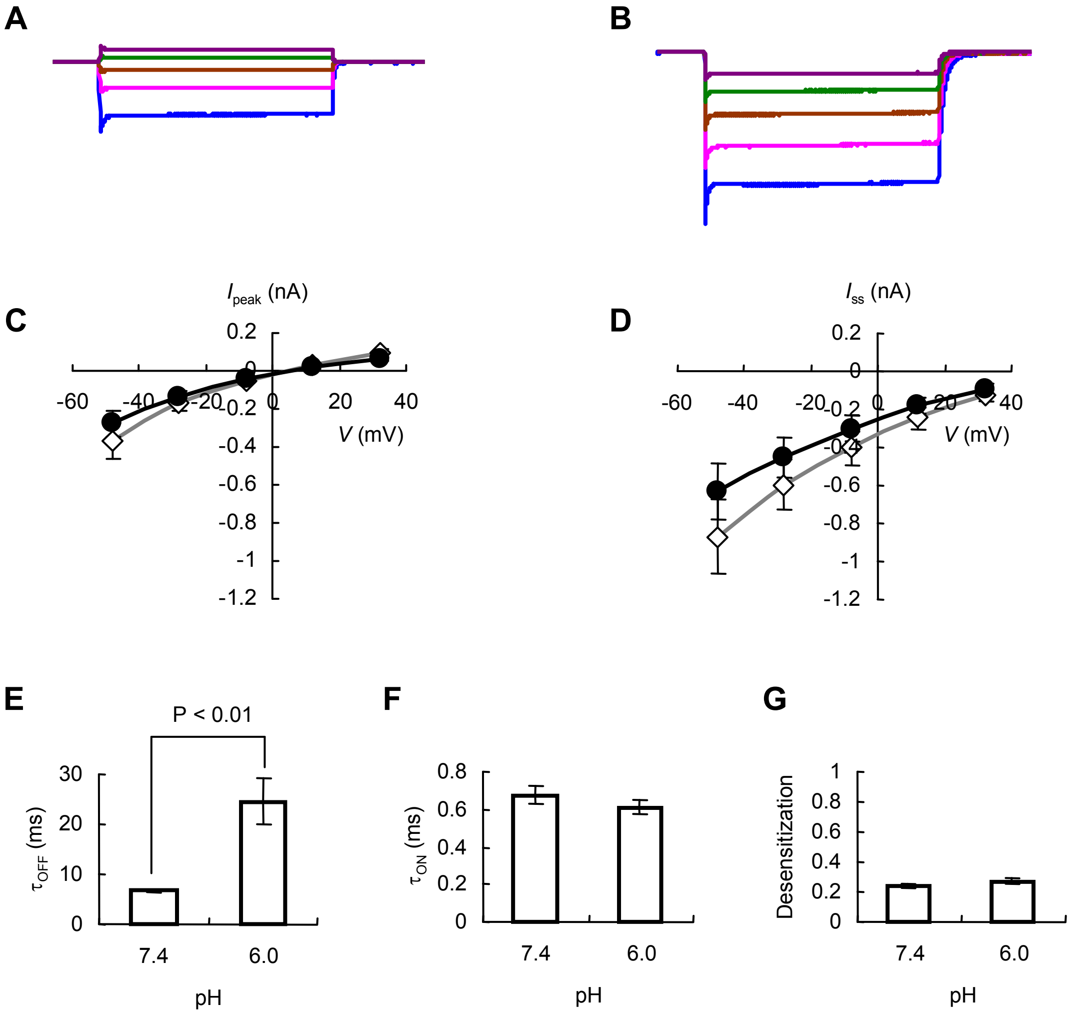

Figure S7. Sensitivity to pH. A and **B**. ChRGR (ChR1-fg_2) was expressed in a HEK cell and its photocurrent was measured at each membrane potential of -48 (blue), -28 (magenta), -8 (brown), 12 (green) or 32 mV (purple). The pH of external solution was adjusted to 7.4 (A) or 6.0 (B). **C** and **D.** The peak currents (I_{peak}, open diamond) and the steady-state photocurrents (I_{ss}, closed circle) as functions of membrane potential (V). pH 7.4 (C) and 6.0 (D). **E.** The pH sensitivity of τ_{OFF}. The difference was significant ($P < 0.01$, Mann-Whitney *U*-test). **F.** The pH sensitivity of τ_{ON}. **G.** The pH sensitivity of desensitization.
